# Novel Targeted Therapies for Metastatic Thyroid Cancer—A Comprehensive Review

**DOI:** 10.3390/cancers12082104

**Published:** 2020-07-29

**Authors:** Mohammad Al-Jundi, Shilpa Thakur, Sriram Gubbi, Joanna Klubo-Gwiezdzinska

**Affiliations:** Thyroid Tumors and Functional Thyroid Disorders Section, Metabolic Disease Branch, National Institute of Diabetes, Digestive and Kidney Diseases, National Institutes of Health, Bethesda, MD 20814, USA; mohammad.aljundi@nih.gov (M.A.-J.); shilpa.thakur@nih.gov (S.T.); sriram.gubbi@nih.gov (S.G.)

**Keywords:** thyroid cancer, targeted therapy, tyrosine kinase inhibitors, immunotherapy

## Abstract

The knowledge on thyroid cancer biology has grown over the past decade. Thus, diagnostic and therapeutic strategies to manage thyroid cancer are rapidly evolving. With new insights into tumor biology and cancer genetics, several novel therapies have been approved for the treatment of thyroid cancer. Tyrosine kinase inhibitors (TKIs), such as lenvatinib and sorafenib, have been successfully utilized for the treatment of radioactive iodine (RAI)-refractory metastatic differentiated thyroid cancer (DTC). In addition, pretreatment with mitogen-activated protein kinase (MAPK) inhibitors (trametinib and selumetinib) has been shown to restore RAI avidity in previously RAI-refractory DTCs. Local therapies, such as external beam radiation and radiofrequency/ethanol ablation, have also been employed for treatment of DTC. Vandetanib and cabozantinib are the two TKIs currently approved by the Food and Drug Administration (FDA) for the treatment of medullary thyroid cancer (MTC). Other novel therapies, such as peptide receptor radionuclide therapy and carcinoembryonic antigen (CEA) vaccine, have also been utilized in treating MTC. Ongoing trials on selective rearranged-during-transfection (RET) protooncogene inhibitors, such as LOXO-292 and BLU-667, have demonstrated promising results in the treatment of metastatic MTC resistant to non-selective TKIs. The FDA-approved *BRAF*/*MEK* inhibitor combination of dabrafenib and trametinib has revolutionized treatment of *BRAF^V600E^* mutation positive anaplastic thyroid cancer. Several other emerging classes of medications, such as gene fusion inhibitors and immune checkpoint inhibitors, are being actively investigated in several clinical trials. In this review, we describe the molecular landscape of thyroid cancer and novel targeted therapies and treatment combinations available for the treatment of metastatic thyroid cancer.

## 1. Introduction

Thyroid cancer is predicted to affect 52,890 new patients in the USA in 2020, with the incidence being three times higher in women as compared with men [1]. Until recently, thyroid cancer was the most rapidly increasing cancer in the USA, largely, but not solely, due to increasing use of sensitive diagnostic procedures. However, the increase of about 7% per year during the 2000s has slowed to 2% per year in men, and rates have stabilized in women from the period of 2012 to 2016, as a result of the more conservative diagnostic approach that has been recently implemented [1]. Differentiated thyroid cancer (DTC) that arises from follicular cells is further subclassified into papillary thyroid cancer (PTC), which is the most common histological type, follicular thyroid cancer (FTC), and Hürthle cell cancer (HTC). De-differentiated thyroid cancer is classified as poorly differentiated thyroid cancer (PDTC) and anaplastic thyroid cancer (ATC). Medullary thyroid cancer (MTC) arises from neuroendocrine C cells that are derived from the neural crest [2].

The standard of care for DTC consists of surgery (lobectomy or total/near-total thyroidectomy, with or without lymph node dissection), dependent on the tumor size, extrathyroidal extension, and metastatic potential. Very low-risk PTCs can be serially followed with active surveillance, using neck ultrasound (US) without surgical intervention, if the size remains stable over time [3].

Following surgery, all patients classified as high-risk for persistent/recurrent disease with evidence of radioactive iodine (RAI)-avid metastases, as well as patients classified as intermediate risk for persistence or recurrence, receive RAI treatment, as it has shown to decrease mortality and/or morbidity, respectively [3,4]. Metastatic RAI-non-avid disease carries a worse prognosis, as the five-year survival is estimated to be as low as 10% [3].

In contrast to DTC, all patients with MTC undergo total thyroidectomy with or without lymph node dissection. Patients with MTC do not benefit from RAI therapy, as the neuroendocrine tumors lack the sodium-iodide (NIS) symporter necessary to incorporate RAI within the cell [5]. External beam radiation to the neck may be used for persistent or residual local disease [5]. The overall 10-year survival for MTC confined to the thyroid gland is 95.6% but is as low as 40% for patients with distant metastatic disease at the time of diagnosis [6].

Since standard RAI therapy for DTC is not effective in 5–22% of patients, and surgical treatment is not curative in patients with MTC presenting with metastatic disease, small molecules have been developed that target aberrant signaling pathways specifically found in thyroid cancer [3]. Therefore, understanding of the molecular landscape of thyroid cancer is crucial to provide individualized targeted therapies.

## 2. Molecular Landscape of Thyroid Cancer

Dysregulation of multiple signaling pathways, such as mitogen-activated protein kinase (MAPK), phosphoinositide-3-kinase (PI3K), receptor tyrosine kinase (RTK), and Wingless/Integrated (WNT), has been reported to contribute toward the pathogenesis of thyroid cancer and is generally associated with the genetic alterations of genes involved in these pathways [7]. The most common driver genetic alterations observed in DTC include mutations in *murine sarcoma viral oncogene homolog B (BRAF), rat sarcoma (RAS)*, and *RET* (rearranged-during-transfection)/*PTC* rearrangements; in PDTC and ATC, *TP53* (tumor protein p53) mutations are often observed; and in MTC, point mutations in *RET* oncogene and pathogenic *RAS* variants are common. These genetic alterations are affecting MAPK and PI3K signaling [8]. In this section, we discuss the molecular alterations that have been identified in different types of thyroid cancers, including DTC (PTC, FTC, and HTC), ATC, PDTC, and MTC.

### 2.1. Molecular Alterations in MAPK and PI3K Signaling Pathway

**Figure 1:**
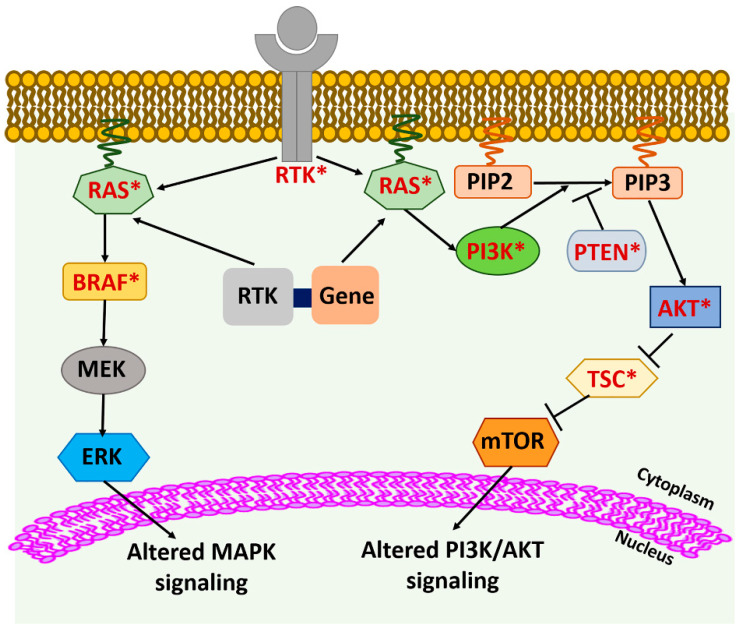
Genetic alterations affecting MAPK and PI3K signaling pathways in thyroid cancer.

The most common genetic alterations associated with MAPK and PI3K signaling pathways in thyroid cancer include mutations in *BRAF, RAS, PTEN (Phosphatase and tensin homolog), AKT (Protein Kinase B)*, and *PI3KCA (Phosphatidylinositol-4,5-Bisphosphate 3-Kinase Catalytic Subunit Alpha)* [7] (Figure 1 and Table 1). *BRAF^V600E^* is the predominant mutation in PTC (18–87%) but has also been detected in PDTC, ATC, and HTC [9,10]. Other rarely detected *BRAF* variants include *BRAF^K601E^* mutation, large insertion/deletions, fusions, and small deletions [11]. *RAS* mutations also have been detected with a prevalence rate of 30–50% in FTC, 30–45% in follicular-variant of PTC (FVPTC), 15% in HTC, 20–40% in PDTC and ATC, and 10–30% in MTC and rarely in classical PTC patients [7,10,12,13,14,15]. The most common *RAS* mutations occur at codons 12, 13, and 61, which are considered mutational hot-spot regions and may affect any of the three *RAS* genes—*HRAS*, *KRAS*, and *NRAS* [16]. The most predominant *RAS* mutation to be detected in thyroid neoplasms is *NRAS61* [17].

*BRAF* and *RAS* mutations are mutually exclusive genetic alterations, suggesting that the presence of either of these genetic variants is sufficient for thyroid tumorigenesis [40] (Figure 1). Based on the differences of the gene profile of the *BRAF^V600E^* and *RAS* mutant tumors, a BRAF^V600E^-RAS score (BRS) has been developed that can categorize PTCs into either *BRAF^V600E^*-like PTCs or *RAS*-like PTCs [11].

Members of the PI3K pathway that are mutated in thyroid cancer include *PTEN, AKT*, and *PIK3CA*. However, the frequency of their presence is low [41] (Figure 1 and Table 1). *PTEN* is a tumor suppressor gene, and mutations, deletions, and epigenetic changes leading to its loss of expression have been observed in DTC, PDTC, ATC, and HTC [7,10,11,42]. Somatic single nucleotide variants (SSNVs) of *AKT1*, *AKT2*, and *AKT3* have been detected in thyroid cancer at a very low frequency [11,43]. An *AKT1* (G49A) mutation has been seen in thyroid cancer metastasis, suggesting that this mutation arises late during progression [44]. Copy number gain of *AKT1* has also been reported in ATC and FTC [21]. The genetic alterations reported in the *PIK3CA* gene in thyroid cancer include gene amplification and, less commonly, mutations [45]. As in *AKT1*, genetic alterations in *PIK3CA* appear late during malignant progression of thyroid cancer and are more common in ATC than DTC [44,45] (Figure 1 and Table 1).

In MTCs, the most common genetic alteration related to MAPK/PI3K signaling pathways is *RAS* mutation. Mutations in *KRAS* and *HRAS* genes have been identified in *RET* wild-type sporadic MTCs and are mutually exclusive events in MTCs [46].

### 2.2. Molecular Alterations in RTK Signaling Pathway

*RTKs* play a crucial role in the regulation of processes such as proliferation, differentiation, and metabolism, and genetic alterations in *RTKs* have been reported frequently in cancer [33]. In thyroid cancer, the *RET* proto-oncogene is one of the most common *RTKs* to be altered. The most prevalent genetic alterations of *RET* in PTC are *RET–PTC* fusions. The *RET/PTC* rearrangements are the predominant genetic alterations in sporadic childhood DTCs and in radiation-related DTCs [47]. The *RET*/PTC1 rearrangement was a common oncogenic alteration to be observed in childhood thyroid cancer cases after the Chernobyl nuclear power plant accident, which resulted in radiation exposures [47]. Moreover, *RET* fusions have been observed in almost 50% of atomic bomb survivors with PTCs who had high radiation exposure [48]. 

Various *RET* fusions have been reported with genes such as *CCCD6* (Coiled Coil Domain Containing 6), *NCOA4* (Nuclear Receptor Coactivator 4), *ERC1* (ELKS/RAB6-Interacting/CAST Family Member 1), *AKAP13* (A-Kinase Anchoring Protein 13), *FKBP15* (FKBP Prolyl Isomerase 15), *SPECC1L* (Sperm Antigen With Calponin Homology And Coiled-Coil Domains 1 Like), *TBL1XR1* (Transducin Beta Like 1 X-Linked Receptor 1), and *TRIM27* (Tripartite Motif Containing 27) [7,11]. The genetic alterations in *RET* can lead to constitutive *RET* activation and stimulation of the MAPK and PI3K signaling pathways [7].

Neurotrophic receptor tyrosine kinase (NTRK) rearrangements have been reported in PTCs. NTRKs are receptor tyrosine kinases encoded by the *NTRK1, NTRK2*, and *NTRK3* genes [11,49,50]. The NTRK fusion proteins are phosphorylated at tyrosine residues, leading to the constitutive tyrosine-kinase activity and stimulation of MAPK, PI3K, and phospholipase C-signaling pathway [7,49,50] (Figure 1 and Table 1).

Mutations and gene rearrangements have also been reported in anaplastic lymphoma kinase (*ALK*) gene in thyroid cancer. The *ALK* gene fusions are mainly observed in ATC and PDTC, but are also present in PTC, at low frequencies [7,51]. Two *ALK* mutations (C3592T and G3602A) have been identified in ATC. These mutations lead to amino acid changes (L1198F and G1201E) within the *ALK* tyrosine kinase domain, resulting in increased tyrosine kinase activities [31]. The *ALK*-associated genetic alterations activate the downstream MAPK and PI3K signaling pathways [7,31] (Figure 1 and Table 1).

Other *RTK* genetic alterations include *FGFR2 (Fibroblast Growth Factor Receptor 2)* gene fusions with *OFD1 (Oral-Facial-Digital Syndrome 1) and VCL (Vinculin)* in PTC [11]; missense mutations in *EGFR (Epidermal Growth Factor Receptor), FGFR2*, and *FLT3 (Fms-Relat.ed Receptor Tyrosine Kinase 3)* in PDTC [32]; and copy number gains in *EGFR, PDGFRα (Platelet-Derived Growth Factor Receptor Alpha), PDGFRβ (Platelet-Derived Growth Factor Receptor Beta), VEGFR1 (Vascular endothelial growth factor receptor 1), VEGFR2 (Vascular endothelial growth factor receptor 2), KIT (KIT Proto-Oncogene)*, and *MET* (MET Proto-Oncogene) in ATC and FTC [21]. In HTC, mutations of various RTK genes *(EGFR, ERBB2-Erb-B2 Receptor Tyrosine Kinase 2, PDGFR, MET*, and *RET*) were observed in 20% of the tumors [10]. 

In MTCs, the most common *RTK* alteration is the *RET* gain of function point mutations in the *RET* oncogene. MTC can be sporadic (70–80% cases) or hereditary (20–30% cases), and about 40–70% of the sporadic and 95% of the hereditary cases harbor mutations in the *RET* oncogene [28,29]. The *RET* mutations associated with MTC occur at the cysteine-rich or tyrosine kinase domains located within the seven exons (8, 10, 11, 13, 14, 15, and 16) [52]. These mutations can lead to one of the three clinically distinctive subtypes of hereditary MTC—multiple endocrine neoplasia (MEN) type 2A (MEN2A), and MEN2B, and familial MTC [53]. The most frequent *RET* point mutation to be detected in 85–90% of MEN2A patients and a small subset of sporadic MTC patients occurs at codon 634 [28,52]. On the contrary, the most commonly observed *RET* mutation in 95% of the MEN2B and 75–95% of the sporadic cases is M918T [54]. To this date, several *RET* mutations have been identified in sporadic and hereditary MTC. It has been shown that different *RET* mutations can produce distinct phenotypes that differ in terms of the aggressiveness of MTC, age of onset of MTC, and the presence/absence of other endocrine malignancies [52]. Based on the genotype–phenotype associations, the *RET* mutations have been stratified into three risk groups (highest risk, high risk, and moderate risk) that reflects the aggressiveness of MTC [55]. The highest risk *RET* mutation is M918T mutation, and the patients with this mutation develop the aggressive form of MTC and metastasis at a very young age [55]. The *RET* mutations that fall in the high-risk group include mutations in codons 634 and 883, and the patients with these mutations are at risk of developing an aggressive form of MTC at an early age [55]. The moderate risk *RET* mutations occur at codons 533, 609, 611, 618, 620, 790, 804, and 891, and the patients with these mutations typically show a less aggressive form of MTC in comparison with the high-risk and highest-risk groups and later onset [55]. Moreover, *RET* and *ALK* gene fusions have also been observed in MTC at low frequencies [51,56].

Receptor tyrosine kinases (*RTKs*) are upstream of the MAPK and PI3K pathways. The mutations in the *RTKs* and *RTK*-gene fusions can alter the downstream *RAS*/MAPK and PI3K/AKT pathways, leading to oncogenic transformation/progression. Similarly, the mutations in the components of the MAPK and PI3K pathways can promote tumorigenesis. Genes that have shown to be mutated in thyroid cancer are shown in red with an asterisk.

### 2.3. Other Molecular Alterations

**Figure 2:**
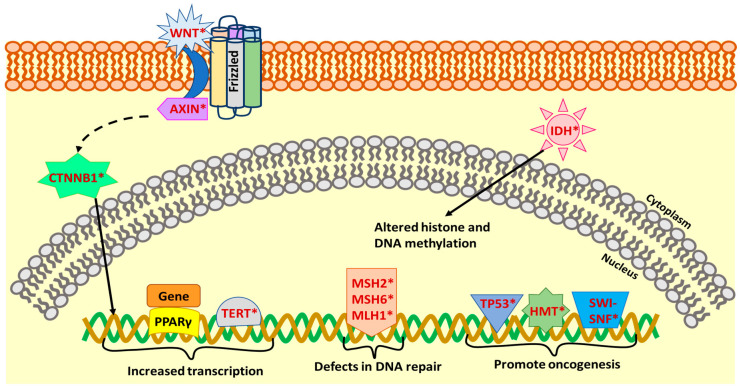
Genetic alterations affecting nuclear processes in thyroid cancer. The figure shows mutations/gene fusions affecting nuclear processes such as transcription, DNA repair, and epigenetic regulation of genes to promote oncogenesis. The genes that have been observed to be mutated in thyroid cancer are shown in red with an asterisk.

*PAX8-PPARγ* (*Paired* Box 8-*Peroxisome Proliferator Activated Receptor Gamma*) rearrangement is present in 30–35% of FTC and 2–13% of follicular adenomas (FA) [36,57]. Besides this, *PAX8-PPARγ* gene fusion also appears with variable frequency in the FVPTC [36,57]. This gene fusion can act as a dominant-negative activator of *PPAR-γ* and can act as a transcriptional activator of a subset of *PPARγ*-inducible genes leading to its oncogenic effects [36] (Figure 2). 

*TP53* mutations leading to the inactivation of this tumor suppressor gene are common in ATC (50–80%) and PDTC (10–35%), but have been detected with variable frequency in FTC and PTC as well [7,35] (Figure 2, Table 1). These mutations usually occur between exons 5–8 and promote tumor development as well as progression [35]. *Telomerase reverse transcriptase (TERT*) promoter mutations are also detected in ATC (40–70%), PDTC (40%), an aggressive form of HTC (32%), PTC (10%) and FTC (20%) [7,10,37], (Figure 2, Table 1). The other common mutations in ATC and PDTC include mutations of the components of the Wnt pathway [34]. This includes the gain of function mutations of *CTNNB1* (β-catenin gene) and loss of function mutations of the *Axin1* gene (Figure 2 and Table 1). The mutations in β-catenin promote its nuclear localization and may increase transcription [34]. The other genetic alterations to be predominantly observed in less differentiated thyroid cancer include mutations in the members of the DNA Mismatch Repair pathway (*MSH2—MutS Homolog 2, MSH6—MutS Homolog 6*, and *MLH1—MutL Homolog 1*); members of the SWI-SNF chromatin remodeling complex; eukaryotic translation initiation factor 1A (*EIF1AX*); histone methyl-transferases (*HMTs*); and isocitrate dehydrogenase 1 (*IDH1*) [7] (Figure 2 and Table 1).

A comprehensive analysis of the genomic landscape of HTC was recently performed which revealed unique genetic alterations in addition to the common genetic alterations that are seen in other types of thyroid cancer [10]. The common genetic alterations in HTC include mutations in mitochondrial genes, epigenetic modifiers, and components of DNA damage and repair pathway; chromosome 5 and 7 duplications; loss of heterozygosity; and in-frame gene fusions [10].

The molecular landscape of thyroid cancer forms a basis for the implementation of targeted therapies. 

### 2.4. Mechanisms of RAI-Refractoriness in DTC

**Figure 3:**
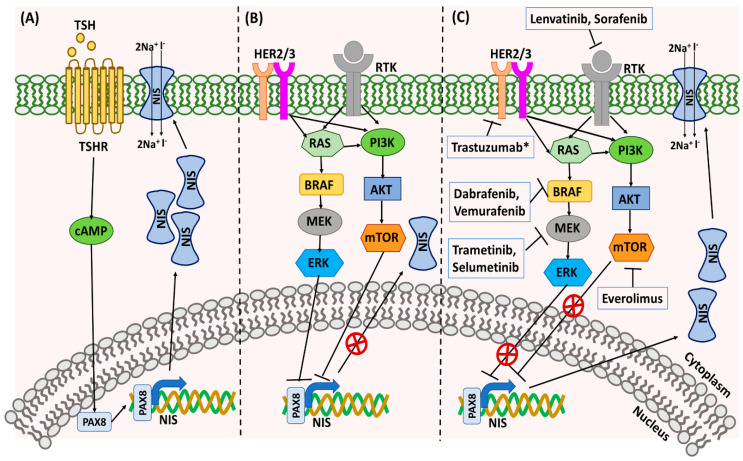
Mechanism of RAI resistance and its reversal with targeted therapies. (**A**) Under normal conditions, expression of sodium-iodine symporter (NIS) is regulated through TSHR, resulting in stimulation of thyroid-specific transcriptional factors such as *PAX8*, which promotes NIS transcription and its expression on the cell surface. (**B**) In thyroid tumor cells with hyperactive MAPK and PI3K/AKT signaling, the NIS transcription is repressed, resulting in loss of its cell surface expression and RAI resistance. (**C**) Targeted treatment with inhibitors that block the MAPK/PI3K signaling pathways improves NIS expression and RAI avidity. * Trastuzumab has been tested in breast cancer model to target HER2 signaling but not in thyroid cancer model.

RAI-refractoriness is the loss of ability of the DTC cells to take up and concentrate radioactive ^131^I, which is necessary to eliminate foci of persistent/recurrent disease [58]. Approximately 5–15% of DTC patients become refractory to RAI by losing the expression of sodium iodide symporter (NIS), which occurs as a result of the loss of thyroid differentiation features [58,59]. There are multiple mechanisms responsible for the loss of NIS expression on the cell membrane of the thyroid cancer cells. In the thyroid, TSH regulates NIS expression through stimulation of TSHR, which activates adenylyl cyclase and results in the accumulation of cyclic AMP (cAMP) within thyroid cells (Figure 3) [60]. This increase in cAMP induces NIS transcription by stimulating thyroid-specific transcriptional factors (TTFs), like paired box 8 (*PAX8*) [59,60]. Studies have suggested that aberrant activation of MAPK/ERK and PI3K/AKT pathways are responsible for the TTF repression and loss of NIS expression (Figure 3) [59]. The presence of *BRAF^V600E^* mutation has been strongly correlated to the loss of NIS expression and RAI refractoriness in PTC patients [61]. BRAF activation has been shown to repress *PAX8* binding to the NIS promoter by activating TGFβ (transforming growth factor β)/Smad3 signaling [62,63]. In addition, activated BRAF can also promote NIS silencing by driving histone deacetylation of the NIS promoter [63,64]. Targeted treatment with BRAF and MAPK inhibitors have been shown to re-sensitize thyroid cancer cells/tumors to RAI therapy by improving NIS expression (Figure 3) [65,66,67].

Activation of the PI3K–AKT pathway has also been correlated to loss of NIS expression. Various growth factors, such as insulin, insulin-like growth factor 1 (IGF-1), hepatocyte growth factor (HGF), or epidermal growth factor (EGF), leading to PI3K activation and AKT phosphorylation, downregulated NIS expression in thyrocytes [68]. Conversely, inhibition of the PI3K pathway promoted NIS expression in rat thyroid cells and human PTC cells [69]. Activation of mTOR—a downstream target of PI3K/AKT pathway—also resulted in decreased NIS expression in thyrocytes, thus suggesting the involvement of the PI3K–AKT pathway in controlling NIS expression in thyroid cells (Figure 3) [68]. Similarly, *RET/PTC* rearrangement has also been reported to reduce NIS expression in thyroid cells [69]. In addition to this, the presence of *TERT* promoter mutations has been correlated to radioiodine refractoriness in distant metastatic DTC patients [70].

### 2.5. Progression from DTC to Advanced Thyroid Carcinomas

The integrative analysis of the genomic landscape of well-differentiated thyroid tumors revealed the presence of a low frequency of somatic alterations, with the majority of those tumors harboring mutually exclusive mutations in *BRAF* and *RAS* genes, as well as gene fusions that primarily involve *RTKs* [38]. The progression of DTC to advanced thyroid carcinomas involves the acquisition of mutational hits in either oncogenes or tumor suppressor genes. The molecular profiling of advanced thyroid carcinomas showed that the presence of mutations is a much more common event in comparison to well-differentiated thyroid tumors [71]. The predominant mutations in the advanced thyroid tumors were in the components of the MAPK and PI3K/AKT pathway [71]. *BRAF^V600E^* mutation was prevalent in advanced PTC, PDTC, and ATC, whereas *NRAS* mutations were common in advanced FTC and PDTC. The predominant tumor suppressor mutation occurred in *TP53* and was prevalent in HTC, ATC, and PDTC [71]. The presence of co-mutations is also a frequent event in advanced thyroid cancers. Concomitant mutations in *RET/PTC, RAS*, and *BRAF* have been observed frequently in advanced PTC and were associated with poor prognosis [72]. In another study, the integrative analysis of aggressive thyroid carcinomas, including ATC and advanced DTC, revealed the frequent presence of *TERT, AKT1, PIK3CA*, and *EIF1AX* mutations in combination with *BRAF^V600E^* and *RAS* mutations in these advanced forms of thyroid cancers [73]. The most common oncogenic duet that has been investigated for its association with aggressive thyroid cancer phenotype is *BRAF^V600E^* and mutated *TERT* promoter. The presence of *TERT* promoter mutations in synergy with *BRAF* mutations has been associated with aggressive thyroid tumor characteristics, which include lymph node metastasis, distant metastasis, tumor recurrence, multifocality, extrathyroidal extension, and patient mortality [74,75,76,77]. The two parts of this oncogenic duet (*BRAF^V600E^* and mutated *TERT* promoter) have been shown to work cooperatively together to promote oncogenesis, leading to aggressive thyroid cancer phenotype [78]. The underlying mechanism involves increased expression of an E26 transformation-specific (ETS) transcription factor, GA-binding protein subunit beta (GABPβ) through *BRAF^V600E^* driven MAPK pathway leading to the formation of GABPα–GABPβ complex, which binds to the mutant *TERT* promoter and increases TERT expression [78]. Besides *BRAF^V600E^*, *TERT* promoter mutations have also been observed to coexist with *RAS* mutations in aggressive thyroid tumors; however, the clinical significance of this oncogenic duet is still not fully understood [37]. It is likely that *RAS* mutations work cooperatively together with *TERT* promoter mutations through the activated PI3K pathway, to promote aggressive tumor characteristics [37], but this needs to be investigated further. 

## 3. Targeted Therapies for Thyroid Cancer

### 3.1. Tyrosine Kinase Inhibitors

#### 3.1.1. DTC

In November 2013, sorafenib was the first tyrosine kinase inhibitor (TKI) to be approved by the Food and Drug Administration (FDA), for the treatment of progressive metastatic DTC refractory to RAI treatment. Sorafenib targets VEGFR 1-3, PDGFR, *RET*, FLT, and c-kit. The approval was based on the results of the DECISION trial, which was a phase III, multicenter, double-blind, placebo-controlled trial conducted in 417 patients with progressive DTC which failed standard treatment. Sorafenib was associated with a significantly longer median progression-free survival (PFS) (10.8 months) when compared to the placebo (5.8 months). The most common side effects encountered in patients treated with sorafenib were hand–foot skin reaction (76.3%), diarrhea (68.6%), alopecia (67.1%), and rash or desquamation (50.2%) [79,80].

In February 2015, a second TKI lenvatinib, which targets VEGFR2, VEGFR3, EGFR, PDGFR, KIT, and *RET*, was approved for the treatment of progressive DTC refractory to RAI, based on the SELECT trial—A phase III, randomized, double-blind, multicenter study involving 261 patients with progressive DTC. Lenvatinib was associated with a longer median PFS of 18.3 months vs. 3.6 months in the placebo group. Of the 20 deaths that occurred on lenvatinib, six were attributed to the treatment itself, as TKIs lead to QT interval prolongation and fatal tachyarrhythmias [81]. Adverse reactions led to dose reductions in 68% of patients receiving lenvatinib and discontinuation in 18% of patients [82]. Given lack of permanent complete remissions after therapy with the FDA-approved agents, the use of alternative TKIs, as well as combination therapies involving TKIs and mTOR inhibitors or immune checkpoint inhibitors, is being evaluated in several ongoing clinical trials [83,84,85,86,87,88,89,90,91,92,93,94,95,96,97,98,99,100,101,102] (Table 2). 

Since *BRAF^V600E^* is one of the most common mutations in PTC, BRAF inhibitors such as vemurafenib or dabrafenib have been implemented in the management of thyroid cancer. Interestingly, a basket trial of vemurafenib in *BRAF^V600E^* mutation-positive tumors showed significant responses in patients with ATC [105]. However, thyroid cancers exhibit primary resistance to RAF kinase inhibition due to reduced negative feedback, and hence a combination of RAF and MEK kinase inhibitors is necessary to effect MAPK pathway inhibition [115] (Figure 1). An open-label phase II study of 16 patients with *BRAF^V600E^*-mutant ATC treated with dabrafenib in combination with the MEK inhibitor trametinib showed a remarkable overall response rate of 69% in patients with ATC, with one patient achieving complete response [106]. The 12-month duration of response, PFS, and overall survival were estimated at 90%, 79%, and 80%, respectively, a phenomenon not previously seen in ATC, which is usually characterized by overall survival of 3–6 months post-diagnosis [116]. This observation has revolutionized the management of this very aggressive cancer and led to the FDA approval of combination therapy with dabrafenib and trametinib in *BRAF^V600E^*-mutant ATC in May 2018 [106].

#### 3.1.2. MTC

Two additional TKIs—vandetanib, which targets VEGFR2, VEGFR3, EGFR, KIT, and *RET*, and cabozantinib, which targets VEGFR2, *RET*, MET, FLT3, and AXL (AXL Receptor Tyrosine Kinase)—have been FDA-approved for the treatment of metastatic progressive MTC. The treatment with the first one was associated with a longer median PFS of 22.6 compared with 16.4 months in individuals exposed to placebo, while the latter led to a PFS of 11.2 months in the treated group vs. four months in the placebo arm in multicenter, randomized, double-blind phase III clinical trials [103,104,117,118]. Again, the side effect profile was similar to other TKIs and included QT prolongation and arrhythmias, severe hypertension leading to reversible posterior leukoencephalopathy syndrome, headaches, hand–foot syndrome, fistula formation, profound fatigue, decreased appetite, nausea, diarrhea, and abdominal pain [104]. In addition, interstitial lung disease and Stevens Johnson syndrome have been associated with use of vandetanib [104]. 

**Figure 4:**
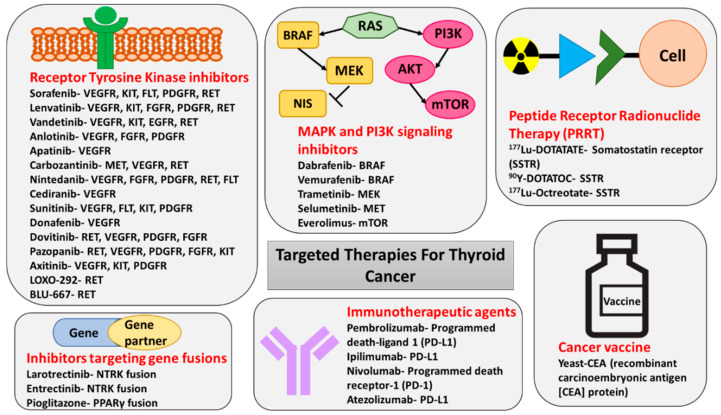
Targeted therapies for the treatment of thyroid cancer. The figure shows inhibitor drug molecules targeting various *RTKs*, components of MAPK and PI3K signaling pathways, and gene fusions in thyroid cancer. The immunotherapeutic agents, PRRT molecules, and a vaccine with the potential for the treatment of thyroid cancer are also included in the figure.

The large number of severe side effects of TKIs result from its ability to target multiple kinases (Figure 4) which play a role not only in cancer progression but also in many important physiological processes. Therefore, more targeted therapeutic strategies have been recently developed and include inhibition of *RET*-only in *RET*-mutated MTCs and PTCs and inhibition of BRAF-only in *BRAF^V600E^*-mutated tumors [106,115]. 

There are two *RET* inhibitors currently implemented in the management of *RET* mutation-positive tumors in the clinical trial setting—BLU-667 and selpercatinib (LOXO-292) (Table 2) [119,120,121,122]. BLU-667 inhibits the protein product of *RETM918T*, as well as *RETV804L/M* gatekeeper mutations conferring resistance to TKIs, while LOXO-292 is a highly selective *RET* kinase inhibitor with nanomolar potency against the canonical *RET* MTC drivers, *RET* gatekeeper mutations, and *RET* fusions [115]. Preliminary data from the ongoing clinical trials documented minimal side effects, excellent tolerability, and high efficacy, with objective response rates (complete and partial responses) ranging from 47% to 62% (Table 2) [115,119,120,121,122].

### 3.2. Therapies Targeting Gene Fusions

An additional development in the management of thyroid cancer which has been proven highly effective in preliminary clinical trials is the use of therapy aimed at targeting *NTRK* and *ALK* gene fusions (Table 2 and Figure 4). Larotrectinib is a selective inhibitor of tropomyosin receptor kinase (TRKA), TRKB, and TRKC which has been approved by the FDA for treatment of solid tumors with NTRK fusion [107,123]. The objective response rate in phase I and II clinical trials was remarkably high (80%) with 63% of patients with NTRK-positive tumors experiencing partial response and 13% complete response [107,123]. Most of the adverse events reported in the primary analysis were grade 1 and grade 2, with the most common being elevated ALT and AST (42%), fatigue (36%), vomiting (33%), dizziness (31%), nausea (31%), diarrhea (29%), and anemia (29%). (Table 2 and Figure 4).

Entrectinib is another selective inhibitor of TRKA, TRKB, and TRKC that also inhibits *ALK* and ROS1 tyrosine kinases. In August 2019, it received FDA approval for treatment of TRK-positive solid tumors, based on results of phase 1 and 2 clinical trials documenting an objective response rate of 57%, including a partial response in 50% and complete response in 7% [108] (Table 2 and Figure 4). The most common adverse events included dysgeusia (43%), dizziness (33%), constipation (33%), diarrhea (28%), and weight increase (26%) [108].

Unfortunately, at the moment, there are no clinical trials targeting THADA or other described above fusions in thyroid cancer, such as *PAX8/PPARγ*, apart from a one case report describing application of pioglitazone in *PPARγ* fusion protein positive metastatic HTC and resulting in the reduction of tumor size and improved pain control [124].

### 3.3. Restoration of RAI Uptake via MEK and BRAF Inhibition

Another important strategy implemented in the management of de-differentiated DTC and PDTC relies on upregulation of sodium iodide symporter NIS via inhibition of its negative regulators MEK and RAF signaling. This strategy re-enables incorporation of iodine within the cancer cell and thus consists of the pretreatment with MEK and BRAF inhibitors, followed by RAI therapy [125,126]. 

The landmark study by Ho et al. has proven the principle that the inhibition of MEK1 and MEK2 by selumetinib induces RAI uptake in RAI-non-avid DTC (Figure 3) [109]. Furthermore, an individualized approach consisting of evaluation of the amount of restored RAI uptake, as measured by ^124^I positron emission tomography/computed tomography (PET/CT)-based tumor dosimetry, has identified patients meeting the tumor accumulation threshold warranting RAI therapy. Among eight patients subsequently treated with RAI, five had a partial response and three had stable disease [109]. Interestingly, these preliminary data suggest a particularly high efficacy of this therapeutic approach in *NRAS*-mutated tumors. Given these promising results, there are several ongoing clinical trials utilizing selumetinib as an adjunct to RAI therapy in larger patients populations (Table 2) [127]. The second-generation MEK inhibitor trametinib is being evaluated in an ongoing phase II trial implementing pretreatment with trametinib, followed by 124I PET/CT-based lesional dosimetry and RAI therapy in patients with sufficiently restored RAI uptake (clinicaltrials.gov identifier NCT02152995) (Table 2). 

Additional clinical trials utilize individualized approaches to patients with *RAS*-mutated and *BRAF*-mutated tumors (Table 2). Patients with tumors harboring an *NRAS* mutations are treated with the MEK inhibitor trametinib, while those with tumors characterized by *BRAF^V600E^* mutation are treated with combination therapies, including BRAF inhibitors such as dabrafenib and vemurafenib and MEK inhibitors (Figure 3). Preliminary data suggest high efficacy of such an approach with partial response to RAI therapy at three months’ follow-up observed in all patients who achieved sufficient restoration of RAI uptake [110]. A similar concept is being utilized in another ongoing multicentric prospective non-randomized phase II trial with two independent arms studying the use of trametinib for *NRAS* mutation-harboring tumors and dabrafenib for *BRAF^V600E^*-harboring tumors (clinicaltrials.gov identifier NCT03244956) (Table 2). 

### 3.4. Peptide Receptor Radionuclide Therapy in Thyroid Cancer

Another therapeutic concept utilizing radiolabeled agents for treatment of metastatic thyroid cancer had been based on a subset of thyroid cancer expresses somatostatin receptors (SSTR) that could be targeted with peptide receptor radionuclide therapy (PRRT) [128,129].

Radiolabeled somatostatin receptor (SSTR) analogs, such as ^68^Ga-DOTATATE, which recognize predominantly SSTR type 2, are utilized for positron emission tomography/computed tomography (PET/CT) imaging. Of note, ^68^Ga-DOTATATE was FDA approved in June 2016 for clinical use for imaging of patients with neuroendocrine tumors, including MTC. Identification of patients with RAI-non-avid DTC and metastatic MTC characterized by positive ^68^Ga-DOTATATE uptake enables identification of individuals who may benefit from PRRT. There were small pilot studies performed in Europe that documented the utility of treatment of progressive RAI-non-avid metastatic DTC and MTC with SSTR agonists radiolabeled with ^177^Lutetium or ^86^Ytrium. The overall response rate was similar to current standard-of-care therapy with TKIs, while the quality of life was better and complication rate was lower after the therapy with PRRT (Table 2) [111,112,113,130,131,132].

### 3.5. Immunotherapy in Thyroid Cancer

#### 3.5.1. DTC

The tumor microenvironment has been acknowledged as the major player in cancer progression and response to therapy. Therefore, the immune landscape of the thyroid cancer has emerged as a potential therapeutic target. Immune checkpoints, such as programmed cell death protein 1 (PD1) and its ligand-PDL-1, as well as cytotoxic T-lymphocyte-associated protein 4 (CTLA-4) inhibitors, exhibit antitumor effects by altering the interaction between the immune system cells and tumor cells [133]. The expression of PD1/PDL1 in thyroid cancer has been extensively studied for both diagnostic and prognostic purposes [134]. In one meta-analysis by Aghajani et al. the expression of PDL1 in thyroid cancer was associated with tumor recurrence and poor survival [135]. Based upon analysis of the TCGA database, a higher level of PD-L1 mRNA was associated with lymph node metastasis, extrathyroidal invasion, and shorter disease-specific survival [136]. The latter was further supported by two independent cohort studies [89,137]. Nevertheless, DTC is thought to be poorly immunogenic due to a relatively low mutation burden. Consistently, published studies have shown that thyroid cancer has relatively poor response to immunotherapy with checkpoint inhibitors [114,138] (Table 2). In order to enhance the efficacy of immunotherapy, combination treatments are being tested in several clinical trials in solid tumors, including thyroid cancer (Table 2). The efficacy of these therapies might be better particularly in de-differentiated tumors, such as widely invasive HTC and ATC, as they are characterized by high mutation burden and higher likelihood of introducing immunogenicity [10,139]. 

#### 3.5.2. MTC

Another interesting concept utilizing the ability of MTC cells to produce carcinoembryonic antigen (CEA) consisted of the introduction of dendritic cell vaccination with anti-CEA vaccine [140,141,142]. Yeast-CEA (GI-6207) is a therapeutic cancer vaccine genetically modified to express recombinant carcinoembryonic antigen (CEA) protein. In a phase 1 trial involving 25 patients with CEA expressing cancers, the treatment with Yeast-CEA showed stabilization of disease and its biochemical biomarkers. It was also well tolerated, with the most common adverse effect being grade 1/2 injection-site reaction [142]. A phase II trial was designed at the National Cancer Institute to evaluate the use of GI-6207 in recurrent medullary thyroid cancer and documented biochemical response without a significant tumor response [143].

## 4. Limitations of Targeted Therapies

The major limitation in applying targeted therapies is its side-effects profile, as well as transient efficacy due to the development of escape mechanisms by the tumors. The first limitation could be overcome by dose de-escalation and adjunct therapy with agents targeting adverse symptoms. The latter requires a thorough investigation of the mechanisms of tumor resistance and analysis of acquired second hit mutations, in order to tailor therapy against changing over time the molecular landscape of the tumor. 

### 4.1. Management of Side Effects of Different Targeted Therapies

#### 4.1.1. Tyrosine Kinase Inhibitors

Systemic treatment with TKIs often leads to side effects affecting different organs and systems. Fatigue is a common side effects that is reported with different agents (Table 2). It can be severe enough (grade 3 and grade 4) to significantly affect the quality of life, leading to interruption or discontinuation of treatment [88,103]. Another common side effect is diarrhea, which can be mild, moderate, or severe. It is thought that it results from the effect of the agent on VEGF and EGF receptors in the bowel [144]. When diarrhea is of grade 1 or grade 2, the treatment is conservative, with antimotility agents such as loperamide and/or diphenoxylate/atropine, as well as cholestyramine and tincture of opium [144,145,146].

The most common cardiovascular adverse event associated with TKIs is elevated blood pressure, which is mediated by the anti-angiogenic effect on VEGF receptors, leading to reduction in endothelial nitric oxide, causing increased vascular tone [147,148]. Given the associated morbidity and mortality from hypertension in these patients, blood pressure should be frequently monitored with a target of <140/90 [148]. If blood pressure is above the target, a treatment with any of the first line anti-hypertensive agent should be initiated [148]. If hypertension is of grade 3 (SBP > 160 and/or DBP > 100) despite optimal treatment with anti-hypertensive agents, then treatment with TKI should be transiently discontinued till blood pressure is at target [148]. Grade 4 hypertension is an indication to stop treatment and consider other treatment options [148]. Other encountered cardiovascular adverse event of TKIs, particularly vandetanib, include QT prolongation, which can cause torsades de pointes ventricular tachyarrhythmia and subsequent cardiac arrest [104]. Therefore, ECG should be routinely done, to monitor QT interval, and QT interval prolongation to >500 milliseconds is an indication to stop treatment [149].

Another organ that can be affected by TKIs is the skin, as muco-cutaneous adverse reactions such as dryness, rash, hyperkeratosis, hand–foot syndrome, alopecia and mucositis occur frequently [150]. Hand–foot syndrome can manifest as hyperkeratosis, skin ulceration, or vesicles and present with pain, tingling, or paresthesia in affected skin areas which, if severe enough, may lead to interruption discontinuation of treatment [151,152]. The management of mucocutaneous side effects relies on preventive measures such as good skin hydration, urea creams, symptomatic treatment such as targeting neuropathic pain with gabapentin, dose reduction, or discontinuation of treatment with TKI [145,150,151].

Some adverse effects are more specific to certain TKIs; for example, pazopanib causes skin and hair hypopigmentation, while sorafenib causes skin hyperpigmentation [79,90]. Treatment with BRAF inhibitors dabrafenib and vemurafenib can be associated with the abnormal skin growth, forming papilloma, keratoacanthoma, nevi, or even skin cancers such as squamous cell carcinoma and melanoma [99,153].

Other encountered adverse effects of antiangiogenic targeted therapies are impaired wound healing, hemorrhage (within the tumor), fistula formation, or viscus perforation (in GI or respiratory tract) [144,154]. Therefore, it is important not to start these agents directly after the surgery for thyroid cancer.

#### 4.1.2. Therapies Targeting Gene Fusion—Tropomyosin Receptor Kinase Inhibitor

The most common side effect of larotrectinib is abnormalities in liver function test [107]. However, transaminitis was rarely severe (grade > 3 or more) and required dose de-escalation only in four patients out 55 involved in the clinical trial targeting NTRK-positive tumors [107]. Other side effects that were less frequent and led to dose de-escalation were neutrophilia and dizziness [107]. Of note, none of the patients who received larotrectinib discontinued treatment due to adverse events [107].

The most frequent adverse events seen with entrectinib were dysgeusia, which was mild and reversible [108]. Other side effects, such as central nervous system related events, weight gain, congestive heart failure anemia, elevated creatinine level, and fatigue, were more severe, requiring dose reduction, treatment interruption, or even discontinuation [108].

#### 4.1.3. Peptide Receptor Radionuclide Treatment

The treatment with (90)Yttrium and (177)Lutetium labeled somatostatin analogs in one study was associated with minor hematological abnormalities, such as leukopenia, erthryocytopenia, and/or decreased hemoglobin level [111]. Most of the hematological abnormalities seen with PRRT are self-limiting, but some patients may develop chronic complications such as myelodysplastic syndrome or leukemia [155]. Elevated liver enzymes, including ALT, AST, GGT, and ALP abnormalities, were also observed [111]. PRRT-related renal toxicity can lead to chronic renal disease/failure especially in patients with comorbidities that affect kidney function, such as hypertension and diabetes [156,157]. This complication is more likely to be observed with 90Ytrium-labeled somatostatin analogs rather than 177 Lutetium radio-labeled. In addition, amino acids infusion before the therapy has reno-protective properties. Unfortunately, amino acids infusion is often associated with nausea and vomiting, which are usually transient [112].

#### 4.1.4. mTOR Inhibitors

A very common side effect of mTOR inhibitor everolimus is stomatitis, which presents as mouth pain, difficulty swallowing, or loss of taste [158]. When severe enough, stomatitis may require stopping oral intake and initiation of parenteral nutrition [158]. Patients receiving stomatitis should be counseled regarding this side effect and encouraged to keep good oral hygiene, to prevent stomatitis [159]. Treatment options include use of mouthwash, topical analgesics, and/or topical corticosteroids [158]. When symptoms of stomatitis are severe and affect the patient, oral-intake treatment should be interrupted till symptoms resolve [158]. In life-threatening situations, everolimus should be permanently discontinued [158].

Another common side effect of everolimus is skin rash, which usually presents as macules and/or papules. This is often associated with pruritis [160]. The mainstay for prevention of skin rash is the use of moisturizers and avoidance of sun exposure [161]. For acne-like rash topical treatment is the first option, but there may be a need to oral antibiotics in severe cases [161].

Other side effects seen with mTOR inhibitors include metabolic derangements such as hyperglycemia and hyperlipidemia, which mandate close monitoring of blood glucose and lipid profile routinely while on everolimus and utilization of anti-hyperglycemic agents and statins for the biochemical control [162].

#### 4.1.5. Immunotherapy

Immune checkpoint inhibitors are associated with immune related adverse effects involving different organs and systems in the human body. Skin is commonly affected by immune checkpoint inhibitors, and the presentation can vary from mild dry skin to life-threatening Steven Johnson Syndrome (SJS) or Toxic Epidermal Necrolysis (TEN) [163,164]. Management of skin manifestation depends on severity; for mild-skin-lesion treatment with antipruritic agents or topical steroids is indicated. Severe manifestations such as SJS require inpatient or even intensive critical care unit admission and treatment with systemic steroids. Grade 4 reactions mandate permanent discontinuation of treatment [164,165]. Another common side effect of immune checkpoint inhibitors is colitis, which often presents as diarrhea. Once other causes of diarrhea are ruled out and the diagnosis is confirmed, treatment can be initiated with oral steroids (budesonide) and antimotility agents [166]. In more severe/refractory cases, treatment interruption or discontinuation and the use systemic steroids or infliximab is indicated [167,168]. Immune checkpoint inhibitors can have different effects on the endocrine system. The effect of immune checkpoint inhibitors on the thyroid gland can lead to hypothyroidism and, less frequently, hyperthyroidism, which is usually not an important consideration in thyroidectomized patients with thyroid cancer [169]. Other less common endocrinological adverse manifestations of immune checkpoint inhibitors are hypophysitis, adrenalitis, and development of type 1 diabetes mellitus [169]. Inflammation of other organs has also been described and includes myocarditis, pneumonitis, hepatitis, nephritis, pancreatitis, and others [170,171,172,173].

## 5. Mechanisms of Tumor Escape from Targeted Therapies

The targeted therapies have shown promising results in the treatment of thyroid cancer, but their biggest limitation is that the tumor cells develop resistance against those therapies over a period of time. The tumor cells acquire resistance to the treatment by developing an escape mechanism against the targeting drugs [174]. Some tumor types have intrinsic resistance to certain drugs; for example, MTCs with *V804M-RET* and *V804L-RET* gatekeeper mutations do not respond to vandetanib treatment. These mutations can also be acquired, over time, upon exposure to vandetanib if they were not already present before treatment [175]. Most of the tumors develop resistance against targeted drugs by acquiring new mutations that result in overactivation of pathways or through induction of alternate pathways to bypass the action of the drug [174].

MAPK and PI3K pathways are the overlapping pathways, and the upregulation of either of these pathways can lead to the same outcome. One possible mechanism by which thyroid tumors overcome the action of multiple kinase inhibitors is through induction of alternate signaling pathways or by upregulating the expression of tyrosine kinase receptors on the cell surface [176]. For example, targeting *RAS* and *RET/PTC* oncogenes in DTC can lead to the induction of PI3K pathway and activation of downstream targets, such as mTOR, forkhead family of transcription factor (FoxO), and others, thus helping tumors escape the drug action [176].

Thyroid tumors acquire RAI resistance by multiple mechanisms, and not all of them are well understood. One of the mechanisms by which thyroid tumors acquire RAI resistance is through the upregulation of the human epidermal receptor (HER) family of receptor tyrosine kinases [176]. HER2 and HER3 are the members of the EGFR family and are upstream of both the MAPK and PI3K pathways [176]. Preclinical data have shown that BRAF-mutant cells escape from the vemurafenib (BRAF inhibitor) effects by overexpressing HER2 and HER3 receptors, leading to the activation of mTOR and MAPK signaling pathways [63,176]. The tumor escape can be overcome by the combination treatment of vemurafenib and HER2 inhibitor trastuzumab, to improve RAI sensitivity (Figure 3) [63].

*ALK* fusion proteins also help thyroid tumors to escape from the effects of TKIs. *ALK* fusions are primarily seen in aggressive thyroid-cancer types, and they are known to activate MAPK and PI3K signaling pathways [177]. *EML4-ALK* fusion and various other *ALK* translocations have been identified in RAI resistant DTC [63]. *STRN-ALK* fusions have been observed in up to 4% ATC and 9% PDTC cases [176]. Combining TKIs with *ALK* specific inhibitors such as crizotinib will be beneficial to patients with *ALK*-positive tumors [63]. 

Thyroid tumors can escape targeted therapies by multiple mechanisms, and to this date, we have very little understanding of those pathways. The biggest challenge in overcoming the tumor escape mechanisms is to identify the mutations or alterations that play a role in the development of resistance against targeting drugs. Identification of the tumor escape mechanism will be helpful in the development of alternative therapies that can be combined with standard therapies to overcome the development of resistance against targeted therapies.

## 6. Conclusions

Several novel targeted therapies have recently been approved by the FDA for use in advanced thyroid cancer. There are many ongoing national and multi-national studies implementing targeted therapies in thyroid cancer. There is a shift in the management paradigm, with the molecular landscape rather than histology/morphology driving an individualized treatment approach. Even amongst the same histological groups of thyroid cancer, the response to therapy is different based on genetic and immune signature of the tumor, further confirming that “one size does not fit all”. The use of the therapeutic agents should be individualized and based on shared decision-making after informing the patient about the possible benefits and disadvantages of the therapy. Encouraged by the results of individualized therapies documenting even complete responses when targeting driver mutations, the aim of future research should focus on finding efficacious treatments that will have a long-lasting curative effect.

## Figures and Tables

**Table 1:** Molecular alterations in thyroid cancer.

Pathways/Cell Components	Genes	Type of Molecular Alterations	Type of Thyroid Cancers (Frequency)	Role in Thyroid Cancer
MAPK and PI3K/AKT signaling pathway	*BRAF*	Activating mutations	PTC (40–80%),	Promotes aggressive tumor phenotype and is associated with poor clinical prognosis and higher risk of recurrence [9,18].
			PDTCs (5–35%),	
			ATCs (10–50%) [7]	
	*RAS*	Activating mutation	FTC (30–50%),	Promotes early tumorigenesis and may predispose DTC to de-differentiate into aggressive forms of thyroid cancer [17,19,20].
			FVPTC (30–45%),	
			HTC (15%),	
			PDTC (20–40%),	
			ATC (10–20%),	
			MTC (10–30%),	
			classical PTC (rarely) [7,10,12,13,14,15]	
	*PTEN*	Inactivating mutation, inactivating deletion, epigenetic changes leading to loss of expression	PTC (2%)	PTEN loss promotes thyroid cancer progression and invasion [23].
			FTC (2–14%),	
			HTC (4–17%),	
			ATC (11–15%),	
			PDTC (4%) [7,10,21,22]	
	*AKT1*	Copy number gain and activating mutations	ATC (19%),	Promotes thyroid tumorigenesis and progression [24,25].
			FTC (8%),	
			PDTC (19%) [7,21]	
	*PIK3CA*	Gene amplification and activating mutations	ATC (18%), FTC (1%),	Promotes oncogenic transformation [26].
			PDTC (2%) [7]	
RTK signaling pathway	*RET-PTC*	Activating gene fusions	PTC (5–25%) [7]	Promotes oncogenic transformation [19,27]
	*RET*	Activating point mutations	Hereditary MTC (95%),	Promotes oncogenic transformation [28].
			Sporadic MTC (40–70%) [28,29]	
	*NTRK*	Gene fusions	PTC (5–25%) [30]	Biological consequence of NTRK fusions in thyroid cancer remains to be explored.
	*ALK*	Activating gene fusions	ATC, DTC [7]	Promotes thyroid cancer progression and aggressiveness [7].
	*ALK*	Activating mutations	ATC (11%) [31]	Promotes thyroid tumorigenesis via anchorage-independent growth, and cell invasion in in vitro model [31].
	Other *RTKs*	Activating gene mutations	PDTC, HTC [10,32]	Activate transcriptional pathways involved in regulation of various cellular processes [33].
		Copy number gains	ATC, FTC [21]	
Wnt pathway	*CTNNB1*	Gain of function mutations	ATC (60–65%),	Involved in thyroid cancer progression [34].
	*Axin1*	Loss of function mutations	PDTC (25%) [7]	
Tumor suppressor	*TP53*	Inactivating mutations	ATC (50–80%),	Promotes tumor progression [7].
			PDTC (10–35%),	
			DTC (variable frequency) [7,35]	
Transcription factors	*PAX8-PPARγ*	Gene fusions	FTC (30–35%),	Promotes oncogenesis by transcriptional activation of downstream effector genes [36].
			FA (2–13%) [7]	
Enzyme	*TERT*	Activating promoter mutations	ATC (40–70%),	Associated with aggressive thyroid cancer phenotype and poor prognosis [37].
			PDTC (40%),	
			HTC (32%),	
			PTC (10%),	
			FTC (20%) [7,10,37]	
DNA Mismatch Repair pathway	*MSH2*, *MSH6*, and *MLH1*	Loss of function mutations	PDTC (2%),	Defective mismatch repair pathway promotes tumorigenesis [38]
			ATC (12%) [38]	
Other pathways/cell components	SWI-SNF chromatin remodeling complex	Nature of mutations remain to be explored	PDTC (6%),	Biological consequence of these mutations in thyroid cancer remain to be explored.
			ATC (36%) [38]	
	*EIF1AX*	Loss of function mutations	PDTC (11%),	Leads to defects in the protein translation [7].
			ATC (9%), PTC (1–2%) [7]	
	Histone methyltransferases (*HMTs*)	Nature of mutations remain to be explored	PDTC (7%),	Biological consequence of these mutations in thyroid cancer remain to be explored.
			ATC (24%) [38]	
	*IDH1*	Probably inactivating mutations	ATC (11%), FTCs (5%) [7]	Associated with loss of IDH1 enzymatic activity, but the tumorigenic role of these mutations in thyroid cancer remain to be explored [7,39].
	Mutations in mitochondrial genes, epigenetic modifiers, and components of DNA damage and repair pathway; chromosome 5 and 7 duplications; loss of heterozygosity; and in-frame gene fusions	Nature of mutations remain to be explored	HTC [10]	Biological consequence of these mutations in thyroid cancer remain to be explored.

**Table 2:** Novel targeted therapies for thyroid cancer treatment.

Drug/ ClinicalTrials.gov ID/Reference	Mechanism of Action	Enrolled Patients *	Primary Outcome	Study Design	Results	Reported Adverse Events
**Tyrosine Kinase Inhibitors**						
Anlotinib Sun et al. [83]	VEGFR, PDGFR, FGFR1	MTC: 58 (locally advanced or metastatic)	PFS	Phase II, single arm, open label	PR: 56.9% PFS at 48 weeks: 85.5% Drop of calcitonin and CEA > 50% in 78% of patients	Hand–foot syndrome, lipid profile abnormalities, fatigue, diarrhea, proteinuria
Anlotinib Li et al. [84]		MTC: 91 (no previous exposure to antiangiogenic agents)	PFS	Phase II/III, two arms, randomized, double blinded, placebo controlled (ALTER0103)	Median PFS: 20.67 months in Anlotinib arm vs. 11.07 months in placebo arm	
Anlotinib NCT04309136		DTC/MTC (locally advanced thyroid cancer with/without distant metastasis)	ORR	Phase II, single arm, open label, recruiting	N/A Estimated End Date 6/2022 (clinicaltrials.gov)	
Anlotinib NCT02586337		DTC (RAI refractory disease)	PFS	Phase II/III, two arms, randomized, double blinded, placebo controlled, active, not recruiting (ALTER01032)	N/A Estimated End Date 12/2019 (clinicaltrials.gov)	
Apatinib NCT03048877	VEGFR	DTC (locally advanced or metastatic disease)	PFS	Phase III, two arms, Randomized, double blinded, placebo controlled, active, not recruiting	N/A Estimated End Date 6/2021 (clinicaltrials.gov)	Hypertension, hand–foot syndrome, proteinuria, fatigue
Apatinib NCT03167385		DTC (locally advanced or metastatic disease)	Disease control rate	Phase II, single arm, open label, recruiting	N/A Estimated End Date 12/2020 (clinicaltrials.gov)	
Axitinib Cohen et al. [85]	VEGFR, PDGFR, KIT	DTC: 45 (resistant to or not appropriate for RAI) MTC: 11 ATC 11	ORR	Phase II, single arm, open label	ORR of 30% SD for ≥ 16 weeks: 38% PFS: 18.1 months	Fatigue, diarrhea, nausea, anorexia, hypertension, stomatitis
Axitinib Capdevila et al. [86]		DTC: 34 (RAI refractory) MTC: 13	ORR	Retrospective study, compassionate use program (CUP) in Spain	ORR for PTC: 29.4% PFS for PTC: 7.4 months ORR for MTC: 23.1% PFS for MTC: 9.4 months	
Cabozantinib Elisei et al. [103]	VEGFR, *RET*, MET, FLT3, AXL	MTC: 219 (locally advanced, metastatic with radiographic progression in the past 14 months)	PFS	Phase III, randomized, double blinded, placebo controlled	PFS: 11.2 vs. 4.0 months for placebo (regardless of *RET* mutation status) PR: 28% (regardless of *RET* mutation status)	Diarrhea, hand–foot syndrome, weight loss, decreased appetite, nausea, fatigue
Cabozantinib NCT03690388		DTC (RAI disease that progressed after use of VEGFR–TKI therapy)	PFS ORR	Phase III, two arms, randomized, double blinded, placebo controlled, recruiting	N/A Estimated End Date 12/2022 (clinicaltrials.gov)	
Cabozantinib NCT02041260		DTC (RAI refractory disease with radiographic progression in the past 14 months)	Number of AEs	Phase II, single arm, open label, active, not recruiting	N/A Estimated End Date 3/2021 (clinicaltrials.gov)	
Donafenib NCT03602495	VEGFR	DTC (RAI refractory/resistant disease)	PFS	Phase III, two arms, randomized, double blinded, placebo controlled, recruiting	N/A Estimated End Date 3/2021 (clinicaltrials.gov)	Hand–foot syndrome, diarrhea, rash, hair loss, hypertension, tachycardia
Dovitinib Lim et al. [87]		DTC: 28 (RAI refractory or not appropriate) MTC: 12	ORR	Phase II, single arm, open label	ORR: 20.5% Median PFS: 5.4 months	Diarrhea, anorexia, nausea, vomiting, fatigue
Lenvatinib Schlumberger et al. [81]		DTC: 261 (RAI refractory progressive disease)	PFS	Phase III, two arms, randomized, double blinded, placebo controlled	Median PFS: 18.3 vs. 3.6 for placebo ORR: 64.8% (CR 4/261 + PR 165/261)	Hypertension, diarrhea, fatigue, anorexia, weight loss, nausea
Lenvatinib Schlumberger et al. [88]	VEGFR, PDGFR, EGFR, *RET*, KIT	MTC: 59 (unresectable progressive disease)	ORR	Phase II, single arm, open label	PR 36%	
Lenvatinib Tahara et al. [89]		Enrolled all types of thyroid cancer, but results reported one cohort for 17 patients with ATC	Serious/non-serious AE	Phase II, single arm, open label	Most frequent AE (Decreased appetite, 82%; HTN, 82%; Fatigue, 59%; Nausea, 59%; Proteinuria, 59%) Secondary Endpoints: ORR: 24% Median PFS: 7.4 months Median OS	
Lenvatinib NCT03573960		DTC (locally recurrent or metastatic progressive RAI refractory disease)	-Grade 3 and higher TEAEs -Number of dose reductions -Time to 1st dose reduction	Phase IV, single arm, open label, recruiting	N/A Estimated End Date 12/2020 (clinicaltrials.gov)	
Lenvatinib NCT03506048		DTC (progressive despite RAI in the past 12 months)	Time to progression	Phase II, single arm, open label, recruiting	N/A Estimated End Date 1/2021 (clinicaltrials.gov)	
Lenvatinib NCT02702388		DTC (RAI refractory disease)	-PFS -TEAEs	Phase II, two arms, randomized, double blinded, evaluating the starting dose 18 mg vs. 24 mg, active, not recruiting	N/A Estimated End Date 9/2020 (clinicaltrials.gov)	
Lenvatinib NCT02966093		DTC (RAI refractory disease in China)	PFS	Phase III, two arms, randomized, double blinded, placebo controlled, active, not recruiting	N/A Estimated End Date 4/2021 (clinicaltrials.gov)	
Nintedanib NCT01788982	VEGFR, PDGFR, FGFR, *RET*, FLT,	DTC MTC (as second-line therapy if progressive disease after first-line therapy)	PFS	Phase II, two arms, randomized, double blinded, active not recruiting	N/A Estimated End Date 9/2019 (clinicaltrials.gov)	Diarrhea, nausea, vomiting, abdominal pain
Pazopanib Bible et al. [90]	VEGFR, FGFR, PDGFR, *RET*, KIT	DTC: 37 (progressive RAI refractory disease)	Tumor response rate	Phase II, two arms, open label	PR: 49%	Fatigue, skin and hair hypopigmentation, diarrhea, nausea
Pazopanib Bible et al. [91]		MTC: 35 (advanced or metastatic disease)	Tumor response rate	Phase II, two arms, open label	PR: 14.3% PFS: 9.4 months OS: 19.9 months	
Pazopanib Bible et al. [92]		ATC: 15 (advanced or metastatic disease)	Tumor response rate	Phase II, two arms, open label	No response	
Sorafenib Brose et al. [79]	VEGFR, PDGFR, *RET*, KIT, FLT	DTC: 209 (locally advanced or metastatic RAI refractory disease)	PFS	Phase III, two arms, randomized, double blinded, placebo controlled (decision trial)	PFS: 10.8 months vs. 5.8 for placebo (regardless of mutation status) PR: 12%	Hand–foot skin reaction, diarrhea, alopecia, skin rash or desquamation
Sorafenib Lam et al. [93]		MTC: 16 (locally advanced or metastatic, arm A—Hereditary MTC, arm B—Sporadic MTC)	ORR	Phase II, single arm, open label	PR: 6.3% SD: 87.5%	
Sorafenib Capdevila et al. [94]		DTC: 16 MTC: 15 ATC: 3 (metastatic progressive unsuitable for surgery, RAI, or radiotherapy)	ORR	Retrospective, Spanish off-label-sorafenib-use program	DTC PR: 19% MTC PR: 47% ATC PR: 33%	
Sunitinib Bikas et al. [95]	VEGFR, PDGFR, *RET*, KIT, FLT	DTC: 23 (metastatic, residual, recurrent, or progressive disease)	ORR	Phase II, single arm, open label, as adjunctive treatment	PR: 26% SD: 57%	Cytopenia, diarrhea, fatigue, hand–foot skin reaction, nausea, musculoskeletal pain, hypertension
Sunitinib Carr et al. [96]		DTC: 28 (RAI refractory disease, FDG–PET avid disease) MTC: 7 (FDG–PET avid disease)	ORR	Phase II, Single Arm, Open Label	DTC ORR: 28% MTC ORR: 50%	
Sunitinib Ravaud et al. [97]		DTC: 41 (RAI resistant) MTC: 26 ATC: 4 (sunitinib as a first-line anti-angiogenic therapy)	ORR	Phase II, single arm, open label	DTC PR: 22% MTC PR: 38.5% ATC: no response	
Vandetanib Wells et al. [104]	VEGFR, EGFR, *RET*, KIT	MTC: 231 (unresectable locally advanced or metastatic disease)	PFS	Phase III, two arms, randomized, double blinded, placebo controlled	PFS HR 0.46 compared to placebo (predicted median PFS 30.5 vs. 19.3 months for placebo) PR 45%	Diarrhea, rash, nausea, hypertension, headache
Vandetanib Leboulleux et al. [98]		DTC: 72 (locally advanced or metastatic disease)	PFS	Phase II, randomized, double blind, placebo controlled	PFS 11.1 vs. 5.9 months for placebo	
Vandetanib NCT01876784		DTC: 119 (locally advanced or metastatic RAI refractory or unsuitable disease)	PFS	Phase III, two arms, randomized, double blind, placebo controlled	PFS (no statistically significant difference—10.0 months vs. 5.7 months with *p* value 0.080)	
**Selective Ret Inhibitors**						
LOXO-292 (Selpercatinib) NCT03157128	*RET*	MTC (among multiple *RET*-fusion and *RET*-activation-positive solid tumors)	Phase I: maximum tolerated dose (MTD), recommended phase II dose Phase II: ORR	Phase I/II, single arm, open label (LIBRETTO-001 trial), recruiting	N/A Estimated End Date 3/2022 (clinicaltrials.gov)	Fatigue, dyspnea, joint pain, insomnia, abnormal liver enzymes (from proof-of-concept study)
LOXO-292 (Selpercatinib) NCT03899792		MTC PTC (among multiple *RET*-altered solid and CNS tumors in pediatrics)	Phase I: safety Phase II: ORR	Phase I/II, single arm, open label (LIBRETTO-121), recruiting	N/A Estimated End Date 10/2022 (clinicaltrials.gov)	
BLU-667 (Pralsetinib) NCT03037385	*RET*	MTC: 49 *RET* mutant PTC: 5 *RET* mutant (among multiple *RET*-altered solid tumors)	Phase I: maximum tolerated dose, number of patients with TEAEs Phase II: ORR, number of patients with TEAEs	Phase I/II, seven groups in Phase II, open label (ARROW TRIAL), recruiting	MTC ORR: 47% MTC PR: 21/49 MTC CR: 2/49 MTC SD: 25/49 Rapid reduction of calcitonin and CEA levels PTC PR 2 of 4 evaluable Estimated End Date 2/2024 (clinicaltrials.gov)	Constipation, elevated liver enzymes, diarrhea, fatigue, elevated serum creatinine, WBC count decrease, hypertension
**BRAF Inhibitors**						
Dabrafenib Falchook et al. [99]	*BRAF^V600E^*	DTC: 13 (BRAF*^V600E^* mutant disease)	ORR	Subset of phase I study	PR: 29%	Skin papilloma hyperkeratosis, alopecia, fatigue, fever, diarrhea
Vemurafenib Brose et al. [100]	*BRAF^V600E^*	DTC: 51 (unresectable and metastatic RAI refractory *BRAF^V600E^* mutant disease)	ORR	Phase II, parallel assignment, open label	PR 38.5% (VEGFR multikinase inhibitor naïve cohort) PR 27% (prior treatment with VEGFR multikinase inhibitors)	Rash, fatigue, arthralgia
Vemurafenib Hytman et al. [105]		ATC: 7 (multiple BRAF*^V600E^* mutant tumors)	ORR	Phase II, basket trial	PR: 14% CR: 14%	
**BRAF/MEK Inhibitor Combination**						
Dabrafenib and Trametinib Subbiah et al. [106]	Dabrafenib: *BRAF^V600E^* Trametinib: MEK1, MEK2	ATC: 16 locally advanced or metastatic BRAF*^V600E^* mutant disease	ORR	Phase II, single arm, open label	PR: 63% CR: 6%	See Dabrafenib See Trametinib
**Tropomyosin Receptor Kinase Inhibitor**						
Larotrectinib Drilon et al. [107]	TRKI: TRKA, TRKB, TRKC	NTRK harboring solid tumors in pediatrics and adults (thyroid cancer: 5 patients)	ORR	NCT02122913 (Phase I adults, open label) NCT02637687 (Phase I/II, pediatrics, open label) NCT02576431 (Phase II pediatrics and adults, basket study)	PR: 100% (5/5)	Elevated ALT and AST, fatigue, nausea, vomiting, dizziness
Entrectinib Doebele et al. [108]	TRKI: TRKA, TRKB, TRKC *ALK*, ROS1	Locally advanced and metastatic NTRK-fusion solid tumors (thyroid cancer: 4 patients)	ORR Median duration of response	(STARTRK-1): NCT02097810 Phase I (STARTRK-2): NCT02568267 Phase II (ALKA-372-001): EudraCT 2012-000148-88 Phase I	Thyroid cancer PR 50% (2/4)	Dysgeusia, dizziness, constipation, diarrhea, weight gain
**Radioactive Iodine Restoration Treatments**						
Selumetinib Ho et al. [109]	MEK1, MEK2	DTC: 20 (RAI refractory disease—9 BRAF mutation, 5 NRAS mutation)	The percentage of patients with selumetinib-induced increases in iodine uptake in the index tumor	Open label, single arm, treatment with selumetinib, then evaluate by RAI uptake study	Increased I-124 uptake in 12/20 (4/9 with BRAF mutations) (5 of 5 with NRAS mutations) (8/12 reached dosimetry threshold for RAI treatment)	Fatigue, maculopapular rash, elevated liver enzymes, acneiform rash
Selumetinib ISRCTN17468602 (UK)		Locally advanced and metastatic RAI refractory DTC	PFS	Phase II, single arm, treatment with selumetinib, then evaluate by RAI uptake study (SEL-I-METRY Trial)	N/A	
Trametinib with RAI NCT02152995	MEK1, MEK2	DTC: *RAS* mutant or *RAS*/RAF wild-type RAI-refractory recurrent and/or metastatic disease	PFS	Phase II, single arm, open label, recruiting	N/A Estimated End Date 12/2020 (clinicaltrials.gov)	Acneiform rash
Trametinib OR Dabrafenib NCT03244956		DTC RAI refractory with *RAS* (trametinib) or BRAF*^V600E^* (dabrafenib) mutation	ORR	Phase II, two arms, open label, recruiting	N/A Estimated End Date 12/2022	See Trametinib See Dabrafenib
Trametinib/ combination Dabrafenib and Trametinib or Vemurafenib and Cobimetinib Irvani et al. [110]	Trametinib (see above), Dabrafenib (see above), Vemurafenib (see above), Cobimetinib: MEK1, MEK2	DTC: 6 (3 BRAF*^V600E^* positive treated with combination, 3 NRAS-positive treated with trametinib)	Restoration of RAI uptake	Retrospective, cohort study	RAI uptake restoration: *BRAF^V600E^* (3/3), NRAS (1/3) with median follow-up 16.6 months	See Trametinib See Dabrafenib See Vemurafenib Cobimetinib: diarrhea, pyrexia, photosensitivity reaction, abnormal LFT, hyponatremia
**Peptide Receptor Radionuclide Therapy**						
PRRT 90Y-DOTATATE or 177Lu-DOTATATE Budiawan et al. [111]		DTC: 7 MTC: 8 Mixed DTC and MTC: 1 (non-RAI avid and RAI refractory thyroid cancer)	Treatment response Treatment related toxicity	Phase II trial, single arm, open label	PR 18.2% SD 36.4% Median PFS 25 months Mean survival 4.2 years	Mild hematological toxicity, abnormal liver enzymes, mild nephrotoxicity
PRRT (90)Y-DOTATOC Versari et al. [112]		DTC: 41 (RAI negative enrolled in the study 11/41 patients were treated with PRRT)	Treatment response	Phase II trial, single arm, open label	ORR 63% (PR 2/11, SD 4/11)	
PRRT 177Lu-DOTATATE or 90Y-DOTATOC Lapa et al. [113]		DTC: 8 (progressive RAI refractory) MTC: 4	Assess tumor heterogenicity in predicting PFS and OS	Phase II trial, single arm, open label	Mean PFS 221 days Mean OS 450 days	
**mTOR Inhibitors**						
Everolimus Lim et al. [101]	mTOR	Thyroid cancer (all subtypes): 38	Disease control rate (PR + SD > 12 weeks)	Phase II, single arm, open label	PR: 5% (2/38, one PTC patient and one FTC) SD: 76%	Mucositis, anorexia, abnormal liver enzymes, acneiform rash
Everolimus Hanna et al. [102]		DTC: 33 MTC: 10 ATC: 7	PFS	Phase II, single arm, open label	DTC: Median PFS 12.9 months, PR 1/38 MTC: Median PFS 13.1 months, PR 1/10 ATC: Median PFS 2.2 months, PR 1/7	
**Immunotherapy**						
Pembrolizumab Mehnert et al. [114]	PD-1 receptor	DTC: 22 (Refractory to standard therapy, with PD-L1 expression)	ORR	Phase 1b, single arm, open label (KEYNOTE-28 Trial)	ORR: 9% PR: 2/22 (9%) SD: 13/22 (59%) Median PFS (PR+SD): 7 months	Diarrhea, fatigue, pruritis, rash, colitis (grade 3 in one patient)
**Combination Therapies Under Investigation**						
Cabozantinib and Atezolizumab NCT03170960	Cabozantinib (see above), Atezolizumab: PD-L1	Multiple tumors, including DTC that is locally advanced or metastatic	Dose escalation: maximum tolerated Dose Expansion: ORR	Phase I/II, dose escalation/expansion, open label, recruiting	Estimated End Date 12/2021 (clinicaltrials.gov)	N/A
Cabozantinib, Nivolumab, and Ipilimumab NCT03914300	Cabozantinib (see above), Nivolumab: PD-1, Ipilimumab: CTLA-4	DTC (RAI refractory progressive after one prior VEGFR therapy)	ORR	Phase II, single group assignment, open label, recruiting	Estimated End Date 1/2021 (clinicaltrials.gov)	N/A
Cediranib Maleate with or without Lenalidomide NCT01208051	Cediranib: VEGFR, Lenalidomide: CRL4^CRBN^ E3 ubiquitin ligase	DTC (unresectable progressive RAI refractory disease)	Phase I: Maximum tolerated dose Phase II: PFS	Phase I/II, parallel assignment, randomized, open label, active, not recruiting	Estimated End Date 2/2020 (clinicaltrials.gov)	N/A
Lenvatinib and Denosumab NCT03732495	Lenvatinib (see above), Denosumab: RANKL	DTC (RAI resistant with bone metastasis)	Skeletal-related event-free (multiple)	Phase II, single group assignment, open label, recruiting	Estimated End Date 6/2022 (clinicaltrials.gov)	N/A
Lenvatinib and Pembrolizumab (PD-1 Inhibitor) NCT02973997	Lenvatinib (see above), Pembrolizumab: (see above)	DTC (progressive RAI refractory disease)	ORR	Phase II, single group assignment, open label, active not recruiting	Estimated End Date 9/2022 (clinicaltrials.gov)	N/A

Abbreviations: AE, adverse event; ATC, anaplastic thyroid cancer; CEA, carcinoembryonic antigen; CR, complete response; CTLA-4, cytotoxic T-lymphocyte-associated protein 4; DCR, disease control rate; DTC, differentiated thyroid cancer; ECG, electrocardiogram; FDA, US Food and Drug Administration; FTC, follicular thyroid cancer; FGFR, fibroblast growth factor receptor; FTL-3, FMS-like receptor tyrosine kinase-3; HR, hazard ratio; MTC, medullary thyroid cancer; MTD, maximum tolerated dose; N/A, not available; ORR, objective response rate; OS, overall survival; PD, progressive disease; PD-1, programmed cell death protein 1; PDGFR, platelet-derived growth factor receptor PD; PFS, progression-free survival; PR, partial response; PRRT, peptide receptor radionuclide therapy; PTC, papillary thyroid cancer; RAI, radioactive iodine; SD, stable disease; SSTR, somatostatin receptor; TEAEs, treatment emergent adverse events; TKI, tyrosine kinase inhibitor; TRKI, tropomyosin receptor kinase inhibitor; RANK, receptor activator of nuclear factor kappa-Β ligand; *RET*, rearranged during transfection; VEGFR, vascular endothelial growth factor receptor. * For placebo-controlled studies, only the number for patients enrolled under the treatment arm is mentioned.
